# Investigating English as a foreign language learners’ perceptions, emotions, and performance during online collaborative writing

**DOI:** 10.3389/fpsyg.2022.954011

**Published:** 2022-09-14

**Authors:** Fahd Hamad Alqasham

**Affiliations:** Department of English Language, College of Sciences and Arts in Methnab, Qassim University, Buraydah, Saudi Arabia

**Keywords:** Blackboard (BB), English as a foreign language (EFL), collaborative writing, emotion, writing skills

## Abstract

Drawing on the sociocultural approach, this study aims to explore EFL learners’ perceptions toward collaborative writing, and the role that learners’ emotions play as a factor influencing their collaboration and achievements in face to face and Blackboard Chatbox as applied in their EFL classes. A mixed-methods research approach was used with a sample of 58 male students enrolled in writing courses at three levels (Levels 1–3) at the Department of English Language and Translation, Qassim University. Three instruments were used for data collection; a 45-item closed-ended questionnaire, semi-structured interviews, and the learners’ overall performance. The findings shown that most learners have positive perceptions toward studying online through Blackboard, and Blackboard Chatbox. Furthermore, Blackboard Chatbox could provide necessary affordances to facilitate learners’ emotion, which could enhance their collaborative writing. However, no significant difference was observed between learners’ performance in the two models of instruction (Sig. = 0.287). Taken together, the results of the present study enhance current understanding of the role of learners’ emotions in collaborative writing with the use of technology.

## Introduction

The sociocultural theory places emphasis on the role of social interaction and collaborative performance in learning and cognitive development. Learning is not a linear entity but a dynamic one influenced by many factors that are at play simultaneously in the learner’s environment. In the context of foreign language learning, students are expected to indulge in activities which promote communication and social interaction to express their wants and needs (Chen et al., 2020; Caprara and Caprara, 2022; Han et al., 2022). In the sociocultural perspective, much as language is seen as a situated phenomenon, so are learning and teaching. The roots of the sociocultural theory lie in Vygotsky (1978) who propounded that learning is a social phenomenon (occurring as a result of social interactions) and learning success depends equally on the learning environment, teachers, learners, materials, staff and resources. The present study builds on the sociocultural theory to investigate the extent to which learning occurs (seen as a product of the learning tools used) in face to face and online modes of instructions.

Early in 2020, universities in Saudi Arabia started online classes using Blackboard (BB) Collaborate Ultra, a real-time video conferencing tool replete with many functions such as file share, record, manage users and sessions, full-screen reader, support for common actions, whiteboard, etc. (Algethami, 2022). Chatbox (CB), also referred to as Blackboard Chatbox (BBCB), is a synchronous feature of the Blackboard Ñollaborate Ultra, where students and teachers simultaneously communicate through written messages. In the Blackboard Chatbox (BBCB), students can ask questions, send comments, answer teachers’ questions, do exercises, as well as type sentences, paragraphs, and essays in a virtual classroom. According to Tonsmann (2014), this feature provides a great platform for co-learners and teachers to interact both in text and acoustically using the chat feature on a one-to-one basis.

In recent years, online collaborative language learning has come to be widely recognized as a valid substitute to face-to-face (FTF) classroom learning (Zou et al., 2018). Online language learning has been reported to boost learners’ social interaction and facilitate critical thinking and knowledge sharing (Kukulska-Hulme and Viberg, 2018; Zou et al., 2018). However, due to various sociocultural factors that can negatively influence learners’ emotions, enhancing collaboration among language learners remains an important challenge (Järvenoja et al., 2013; Näykki et al., 2014). In order to overcome these contextual constraints and to achieve sustainable knowledge construction and interaction, learners’ negative emotions should be regulated, while positive emotions during interaction in language classroom should be sustained (Järvenoja and Järvelä, 2009; Bakhtiar et al., 2018).

Emotion is a complex construct that evolves from learners’ interpersonal and social interaction regulated and shaped by norms and goals (Imai, 2010; Swain, 2013). Recent research has explored the potential of goals and context to facilitate learners’ engagement in collaborative writing (Li and Zhu, 2013, 2017a; Cho, 2017). This body of work revealed that learners’ engagement in collaborative writing can be enhanced through avoiding negative emotions and focusing on positive emotions. However, while previous studies focused on the process of how learners’ emotions influence collaborative writing, relevant research on the end results of emotion on collaborative writing remains scarce.

To address this gap in the literature, the present study seeks to explore how Saudi EFL learners’ emotions (positive and negative) emerge, guide their engagement and interact in an online collaborative learning environment. More specifically, the researcher investigates how EFL students of College of Arts and Science at Qassim University, Saudi Arabia, used the BB educational platform in their EFL classrooms. The students’ performance with the BBCB was compared to that in the conventional FTF learning environment. The specific focus was on the students’ achievements in writing—the most difficult and challenging skill that students need to master for their learning, career, and daily communication (Bruning and Horn, 2000; Al-Shourafa, 2012; Miftah, 2015; Mantra, 2017; Pertiwi and Drajati, 2018; Gunantar and Transinata, 2019; Bin-Hady et al., 2020; Miftah and Syafii, 2020; Syafii and Miftah, 2020). What makes acquisition of writing skills a particular challenge is that mastering writing requires the development of multiple micro-skills, such as identification of topic statement, writing supporting details, reviewing, editing, and so forth (Ahmed, 2020).

## Literature review

### Collaboration and emotion in a virtual English as a foreign language writing environment

Recent years have witnessed the emergence of various technological platforms to develop learners’ English writing skills (Olson and Olson, 2003; Wong et al., 2015; Kashiwa and Benson, 2018). At the same time, applied linguists have adopted different theories to reach a deeper understanding of the optimal way to enhance English writing skills. Most of these approaches focus on collaboration from a sociocultural perceptive (Barrett et al., 2021), which the present study also adopts. According to Miyake and Kirschner (2014), collaborative learning is a form of social knowledge construction that involves various forms of communication, such as essay writing or problem-solving activities. Collaboration is facilitated by appropriate machine-learning technologies (Koschmann, 2002). L2 learners can participate, both formally and informally, to enhance their language ability and proficiency through online collaboration (Yim and Warschauer, 2017). As argued by Kukulska-Hulme and Viberg (2018), collaboration can help in communication issues with peers, completing interactive tasks, or development of a written project.

However, while there has been extensive research on collaborative writing from the perspective of the sociocultural aspects of technology use (Onrubia and Engel, 2009; Zeng and Takatsuka, 2009; Kessler et al., 2012; Sun and Chang, 2012; Stoddart et al., 2013; Li and Zhu, 2017a,b; Yim and Warschauer, 2017), an important gap in previous research is the lack of studies that would explore psychological constructs in an online collaborative EFL writing (Barrett et al., 2021). Accordingly, learning outcomes and contextual variables that might influence the learners’ collaboration in writing in a virtual environment have received little scholarly attention.

Emotion is one of the core aspects to ensure a successful and sustainable language learning environment (Dewaele and MacIntyre, 2016). According to Csikszentmihalyi (2008), when people push beyond themselves, they experience a sensation of novelty or accomplishment, which enhances their positive emotions, such as enjoyment. Moreover, people usually experience excitement when they strive to achieve an important goal (Dewaele and MacIntyre, 2016). Positive emotions have been argued to be beneficial to language learning and development (Csikszentmihalyi, 2008). For instance, Khajavy et al. (2018) found that willingness to communicate among EFL learners was influenced by their positive emotions. Similarly, Dewaele and Alfawzan (2018) reported the beneficial impact of enjoyment on EFL learners’ performance and achievement. According to Fredrickson (2001, 2003, 2004), positive emotions can broaden people’s instantaneous thought-action capacities, create personal resources, and improve resilience. In contrast, negative emotions, such as anxiety and fear of making mistakes, were reported to have an adverse impact EFL learners’ collaborative writing (Fredrickson, 2004; Dewaele and MacIntyre, 2016; Alkodimi and Al-Ahdal, 2021; Rahimi and Fathi, 2021). In the same vein, Rahimi and Fathi (2021) showed that learners’ anxiety declined when learning in an online space, which resulted in learners increased collaborative writing outcomes as compared to those of FTF students. This suggests the need to explore the role of emotion in online settings, especially in the Saudi context.

### Online learning through Blackboard and Chatbox

Blackboard (BB) can be used in educational contexts not only as a source of online learning, but also as a tool for communication through emails, announcements, discussion forums, and podcasts (Alokluk, 2018). Furthermore, along with providing asynchronous communication to both students and teachers, Blackboard Chatbox (BBCB) can also serve as a synchronous platform where students and teachers communicate through written messages in real time. Accordingly, a great deal of communication and learning-related tasks (e.g., report and assignment submission, announcements, and information dissemination, etc.) can be accomplished using BB (Al-Khresheh, 2021).

Several previous studies explored the use of BB as a tool to enhance EFL learning. For instance, Tonsmann (2014) found that BB provides an effective environment for distance learning. There is also evidence on the active use of BB among EFL students to get the course content and study materials, complete assignments, and communicate with teachers (Alzain, 2021). Furthermore, Martin (2008) found that both teachers and students felt positive toward the experience of using BB. In addition, its use—through active access to learning materials and due to the possibility of immediate feedback—had a positive impact on various learning outcomes (Beard and Harper, 2002; Breen et al., 2003). The use of BB was also reported to encourage students to apply knowledge and exchange their views and experiences (Alzahrani and Aljraiwi, 2017). In a study on faculty members’ perceptions of BB at three different Universities in KSA, El Zawaidi (2014) found that most faculty members considered BB to be easy to use which could assist them in teaching due to its technological features. However, along with reporting the high percentage of positive experiences with BB, Al-Khresheh (2021) also mentioned several issues that could slow down the adoption of this tool. Such issues, as argued by AbuSa’aleek and Shariq (2021), can include students’ preference issues, technical issues, and timing issues, among others.

To date, there has been extensive research on the impact of BB on different learning outcomes (Al-Nofaie, 2020; Almekhlafy, 2020; Elzainy et al., 2020; Almelhi, 2021) while research on BBCB received little attention. For instance, Kirkpatrick (2005) found that BBCB can enhance informal communication between learners and the teacher, and thus, increase learners’ engagement and confidence in the language classroom. However, few studies have explored their impact on students’ EFL writing skills. Similarly, little is known about the role of emotions in the learners’ perceptions of and attitude to BB use in EFL a learning environment. Finally, none of the previous studies have explored the role of emotions in online collaborative writing with BBCB and its influence on students’ achievements.

### Purpose of the present study

To bridge the gaps enumerated upon in the literature, the present study aims to explore the role of learners’ emotions on their perceptions to BB. The second goal is to evaluate the impact of learners’ emotions associated with the use of online technology on collaborative writing, as well as the actual effect of online learning through BBCB on learners’ English writing skills.

### Research questions

(1) What are Saudi EFL tertiary learners’ perceptions on studying collaborative writing though Blackboard?
(2) To what extent can learners’ emotions influence collaborative writing through Blackboard Chatbox?
(3) To what extent can Blackboard Chatbox enhance learners’ achievements as compared to face to face?

## Materials and methods

### Research design

This study adopted a mix of quantitative and qualitative research designs to study the research problems. It collected data from three sources: students’ perceptions via the questionnaire, students’ perceptions through the semi-structure interviews (qualitative data) and students’ performance outcomes through the final results of exams.

### Participants

**FIGURE 1:**
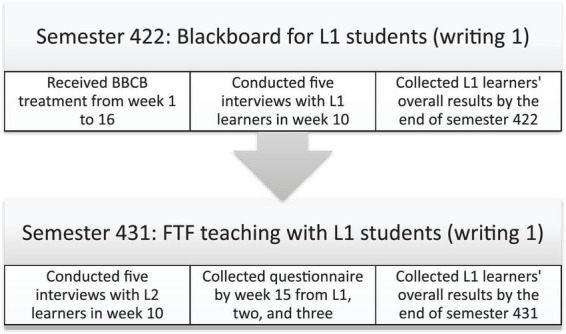
Data collection procedures.

The participants of the present study were 58 undergraduate students enrolled in writing courses from Level 1 to Level 3. They were assigned according to stratified random sampling in which the number of each level is represented according to its total number weights. The specific writing courses in those three levels were Writing-1 (ENG-144), Writing-2 (ENG-145), and Academic Writing (ENG-247). These three courses are taught twice a week, for 1 h and 30 mins each, for a total of 3 h per week. The participants studied in the Department of English Language and Translation, College of Sciences and Arts, Methnab, Qassim University, Saudi Arabia. They came from different cities or villages in the Qassim region. Among the students who answered the questionnaire, 22 students were enrolled in Level One (L1), 17 students studied in Level Two (L2), and 19 students in Level Three (L3). Furthermore, 17 students involved in the experiment were in L1 when they received the treatment in semester 422^1^ (refer to Figure 1). The learners were aged from 18 to 22 years. It may be noted that all the levels were taught through BB in Semester 422.

The instructor who taught the writing courses was an Indian associate professor, with a Ph.D. degree in Applied Linguistics (English), while all the participants were of Saudi origin and native speakers of Arabic. All communication (written or spoken) between the teacher and the students was exclusively in English.

Students’ consent was obtained at the beginning of the semester. The researcher demonstrated to them the objectives of the research and they verbally agreed, this type of approval is workable and valid in the Saudi higher committee for research.

### Data collection instruments

#### Semi-structured interview

For the semi-structured interviews, a set of starter questions were prepared to elicit the students’ perceptions and attitudes to BBCB. The interview questions were face validated by sending them to three university professors in the field of ELT. Their feedback advised that the content be presented in Arabic also. Furthermore, the questions were piloted with three students who were not included in the study. A total of 10 participants were interviewed, and the average length of each interview was 42 mins. In order to ensure optimal comfort level of the participants during the interview and to elicit as much data as possible, all interviews were conducted in Arabic. All interviews were audio recorded, transcribed, and then fully translated into English.

#### Questionnaire

The questionnaire used in this study was developed by the researcher himself keeping in focus the gaps in the literature and the research questions of the present study. Prior to the interviews, the questionnaire was piloted with a different sample from a different class. The validity and reliability of the questionnaire were affirmed by three different experts (two assistant professors and one full professor) in Applied Linguistics. Furthermore, Cronbach’s Alpha was calculated as shown in Table 1. It scored 0.876, which is considered a good measure. The experts’ remarks and suggestions were taken into account to produce the final version of the questionnaire which comprised 45 items distributed across the following three categories: (1) general positive perceptions of BB (15 items); (2) general negative perceptions of BB (10 items); and (3) perceptions of BBCB with regard to enhancing collaborative writing skills (20 items). The questionnaire was created on “Google Forms” and the corresponding link was sent to the students’ WhatsApp groups.

**TABLE 1:** Reliability statistics.

*N* of items	Cronbach’s alpha
45	0.876

#### Learners’ achievements

In order to explore the effectiveness of BBCB in terms of enhancing the participants’ writing skills, the overall results of the learners’ achievements in the midterm and final exams in two semesters were collected (422 and 431). In semester 422, the students in L1 studied using BBCB, while in semester 431, L1 students were taught in the FTF modality. The participants’ achievements were then categorized according to the university evaluation system into nine categories depending on the number of scores (0–100) earned by each student, and corresponding grades (from F to A+) were assigned (see Table 2). Finally, the participants’ results in the two semesters were compared.

**TABLE 2:** Learners’ achievements (scores and grades).

No	Score	Grade
1	95–100	A+
2	90–94	A
3	85–89	B+
4	80–84	B
5	75–79	C+
6	70–74	C
7	65–69	D+
8	60–64	D
9	0–59	F

### Procedures

**FIGURE 2:**
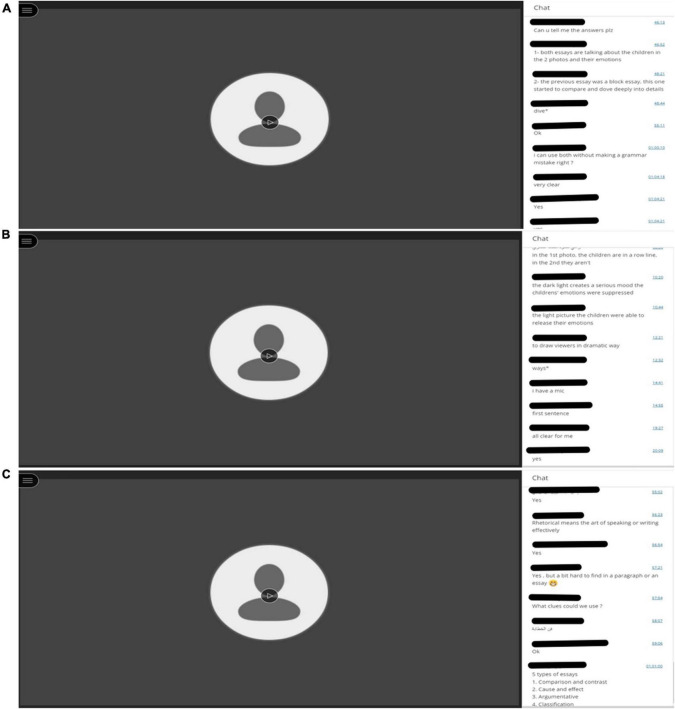
**(A–C)** Student participation in Blackboard Chatbox (BBCB) (picture 1).

The participants were engaged in online learning through BBCB from Week 1 to Week 16. There were no specific rules for the students to follow; they only needed to follow the teacher’s instructions. During the experiment, the students responded to various questions. Figures 2A–C shows a sample of the students’ online learning through BBCB. In addition, as for the data collection procedures, L1 participants received the treatment at first in Semester 422 from week 1 to week 16. Then in week 10 during the same semester, interviews were conducted with 10 participants. Finally, the overall results of the participants’ exams were collected to compare them with those of FTF participants in semester 431 which was the next semester.

In semester 431, a different group of participants were admitted to L1 that received teaching through FTF, the overall results of the new participants of L1 were collected and compared with BBCB participants’ results. By the 10th week of semester 431, interviews were conducted with the same participants as they passed to L2 and received FTF instruction for Writing 2. Finally, by the end of semester 431 the questionnaire was administered and responses collected from L1, 2 and 3 students (see Figure 1, for more clarification).

Students participated in EFL writing activities beginning with a pre-writing stage in which they collected information about the topic they have to write about. They then built on their outlines to suit the nature of the writing tasks. Students sometimes were put into groups of two or three to consolidate cooperative learning. Next, they started with the freewriting task and editing of their writing. Finally, when working with peers, each group’s writing task was submitted to the second group for peer editing before submitting it to the instructor. In fact, each step was timed and this was determined in the writing outline set first. The whole process was given from 30 to 40 mins.

### Data analysis

**FIGURE 3:**
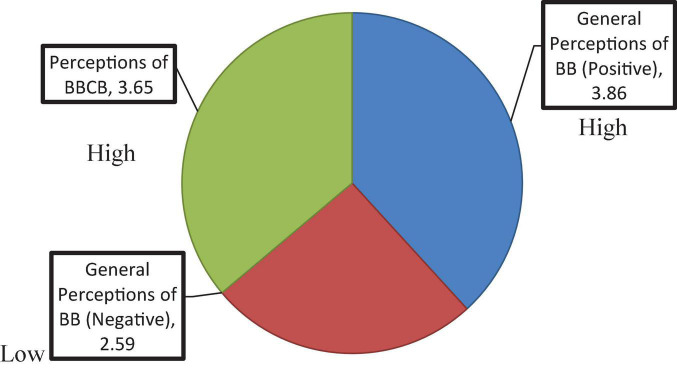
Overall descriptive statistics of student perceptions.

Quantitative data included the participants’ responses to a closed-ended questionnaire and the learners’ overall achievements. The data were analyzed using SPSS (version 22) and mean and standard deviation (SD) values were computed. Furthermore, inferential analysis was performed because the third research question required such analysis to measure the differences between students’ performance in the FTF and BBCB. The results were categorized as follows: very low 1–1.80; low 1.81–2.60; moderate 2.61–3.40; high 3.41–4.20; and very high 4.21–5.00. For an easier comprehension of the overall perception of BB and BBCB use, the means for each of the 45 items were combined and recalculated for each of the following three categories: general perception of BB (Positive), general perception on BB (Negative), and perception on BBCB (see Table 3 and Figure 3).

**TABLE 3:** Overall descriptive statistics of student perceptions.

No	Section	Mean	Range (level)
1	General perception of BB (Positive)	3.86	3.41–4.20 “High”
2	General perception of BB (Negative)	2.59	1.81–2.60 “Low”
3	Perception of BBCB	3.65	3.41–4.20 “High”

Next, qualitative data obtained during semi-structured interviews were analyzed using thematic analysis. To this end, translated transcripts were uploaded to NVivo 12 to find similarities and repeated words. First, several codes were generated; next, these codes were reduced to few major codes that were in line with the theory and quantitative findings. For ethical considerations, all students’ names were replaced with pseudonyms (e.g., Student A).

## Results

In the “Results” section, in Figure 5 the researcher/s compared learners’ achievement in learning through BB and traditional classrooms. Being more of an experimental design, this is explained in detail in the method section with a table (in addition to the figure) presenting the results in the post-test.

### Blackboard

#### Positive perceptions

The first category of items in the questionnaire focused on the learners’ positive perceptions of studying through BB. As can be seen in Table 2 and Figure 3 above, the overall mean of the items in this category was 3.86 which falls in the “high” range (3.41–4.20). Furthermore, Table 4 shows that most learners agreed on the positive aspects of using BB and had positive perceptions of studying through it (mean = 4.27, SD = 0.84; see Table 3). The highest score was obtained for item 9 (“BB saves time”) with a mean value of 4.57 (SD = 0.89), as 69.39% of the study participants strongly agreed and 24.49% agreed on this item. This was followed by item 7 (“BB is easy to use”) and item 15 (“I can access the lecture recordings at any time and get the benefit from them”), with the same mean of 4.46 (SD = 0.89 and 0.95, respectively), where 61.22% and 63.35% of the study participants strongly agreed and 32.65 and 20.41% agreed with this item. The lowest score was obtained for item 1 (“I like to study on Blackboard”), with a mean value of 3.61 (SD = 0.73), where 28.57% strongly disagreed and 22.45% disagreed with the statement.

**TABLE 4:** Students’ positive perceptions of studying through Blackboard (BB).

No	Item	Mean	Std.
1	I like to study on Blackboard	3.61	0.73
7	Blackboard is easy to use	4.46	0.89
9	Blackboard saves time	4.57	0.81
15	I can access the lecture recordings at any time and get the benefit from them	4.46	0.95
	Mean	4.27	0.84

Therefore, questionnaire results clearly indicate the students’ positive attitude toward BB for English learning. Similar conclusions were drawn from the analysis of results of semi-structured interviews. The interviews provided deep information regarding the learners’ positive perceptions that were mainly influenced by their emotions toward the platform. In the interviews, the learners highly valued the use of BB multimodal features which allowed them to easily share their materials with the teacher, which, in turn, enhanced their comfort associated with using the platform and fostered their engagement in the virtual classroom. For example, Student A noted:

*[Positive emotion: Like] There are many good options in the Blackboard system, [Reason: Affordances] you can upload your homework, assignments, send emails, chat, take quizzes, see your attendance and grades, etc., it saves a lot of time (Student A).*

Furthermore, the students positively evaluated the platform’s technological features that facilitated their learning, reduced pressure, and allowed them to save time through not having to physically attend classes, which was an important advantage, as some students live in different cities (located 20 mins to 1 h away from the college). A similar point was made by two other students:

*[Positive emotion: Like] I like blackboard [Reason: Reduced effort] because it saves a lot of time, I don’t have to prepare myself or go for the college and waste a lot of time in the break hours (Students B).*

*[Positive emotion: Like] You can attend all your classes [Reason: Affordances] even if you are sick or you have some other important work to do. Blackboard saved my attendance and I was not debarred from the final exams (Students C).*

Taken together, interview results showed that the students positively evaluated using BB use, as the platform was perceived as comfortable, capable of reducing pressure, and ensuring enjoyment. These findings underscore the importance of using BB in learning environments.

#### Negative perceptions

The second category in the questionnaire was concerned with learners’ problems and negative perceptions and attitudes toward studying through BB. The overall mean of items in this category was 2.59, which falls in the “low” range (1.81–2.60) and indicates that only a few learners had negative perceptions of and attitudes to studying through BB (see Table 2 and Figure 3). Furthermore, Table 5 shows negative perception items that received the highest agreement among the study participants. The highest mean score of 3.40 (SD = 1.38) was given to item 22 (“I like to study in a real classroom rather than on Blackboard”), as 32.65% of the study participants strongly agreed and 14.29% agreed on this statement. The lowest score was obtained for item 23 (“Blackboard is time-consuming”), with a mean value of 1.91 (SD = 0.95), where 2.04% learners strongly agreed and 6.12% agreed on this statement.

**TABLE 5:** Students’ negative perceptions of studying through Blackboard (BB).

No	Item	Mean	Std. deviation
22	I like to study in a real classroom rather than on blackboard	3.40	1.38
23	Blackboard is time-consuming	1.91	0.95
	Mean average	2.655	1.165

Similarly, the analysis of interview data showed that very few participants experienced negative emotions toward BB use. Typical complaints concerned unfamiliarity with the platform, connectivity issues, or a higher risk of getting distracted as compared to the conventional FTF study. For instance, one participant provided the following account:

*[Negative emotion: Worry] Blackboard is just the temporary platform to use and it is not forever; [Reason: Prefers traditional teaching] it has many options that access the work faster, but it cannot be always used for teaching (Students D).*

Along similar lines, another student noted that the platform was boring, and its use can be distracting:

*[Negative emotion: Boring] Classes on blackboard are boring, and there is no seriousness. [Reason: lack of motivation] Learners are engaged in some other work at the same time and it creates disturbance and no seriousness (Students E).*

However, this presumably negative aspect of BB could be bypassed with a proper teaching strategy that would enhance the learners’ engagement. As discussed previously, the lack of experience with using educational technologies in general might have negatively affected perceptions of some learners. In addition, an appropriate preparation of students to the use of technology by the teacher could have increased the learners’ positive perceptions of BB use.

Another negative aspect of using BB mentioned by the study participants was network connectivity. For instance, one of the students shared the following account:

*[Negative emotion: Dislike] For me, I don’t like to study on blackboard [Reason: Constraints] because I come from a village and there is always signal issue and I cannot access the classes due to the bad connectivity, blackboard needs strong internet because there is video, teacher speaks in the microphone and I cannot listen to him. Most of the time I go to another place and attend my classes there (Student F).*

In summary, the results of both quantitative and qualitative data revealed that the participants had predominantly positive attitudes to and experiences with BB. The rare negative evaluations could be remedied by better teacher and student preparation for the use of the platform.

#### Blackboard ChatBox

Both quantitative and qualitative results showed the participants’ positive perceptions of BBCB use to enhance their writing skills. The mean of the items in this category was 3.65, which falls within the “high” range (3.41–4.20; see Table 3 and Figure 3). As shown in Table 6, the highest score was assigned to item 36 (“I watch other learners’ comments and questions in the chat box and learn from them”), which had a mean value of 4.38 (SD = 0.67). This was followed by item 42 (“I feel proud when I see my teachers’ positive comments and feedback on my writing in the Chatbox”), with a mean value of 4.24 (SD = 0.80) and item 32 (“I write questions in the Chatbox when I face problems”), with a mean value of 4.06 (SD = 1.02). Lower ratings in this category were obtained for item 40 (“I can watch recording at any time and correct my mistakes in paragraphs or essays”), with a mean score of 4.06 (SD = 1.24) and item 33 (“I write comments in the Chatbox”), with a mean value of 4.00 (SD = 1.11).

**TABLE 6:** Students’ perceptions of Blackboard Chatbox (BBCB) in enhancing writing skills.

No	Item	Mean	Std.
32	I write questions in the chat box when I face problems	4.06	1.02
36	I watch other students’ comments and questions in the chat box and learn from them	4.38	0.67
40	I can watch recording at any time and correct my mistakes in paragraphs or essays	4.06	1.24
42	I feel proud when I see my teachers’ positive comments and feedback on my writing in the chat box	4.24	0.80
	Mean average	4.185	0.9325

The results of the quantitative analysis were further corroborated by the findings from semi-structured interviews. As illustrated below, many students positively evaluated the immediacy feature through which BBCB effectively enhanced their learning experience:

*[Positive emotion: Comfortable] When I need a further illustration about something [Reason: Affordances] I don’t have to drive all the way to college or wait until the next day. I just ask my question in BBCB, and the teacher will reply (Student A).*

Collaborative learning was also noted by another study participant:

*[Positive emotion: Enhanced by collaboration] Sometimes, when you want to ask a question. [Reason: Collaboration] you find that another student asked it and the teacher replies give details that you could go back to at any time (Student C).*

Furthermore, some students commented that, through the auto correction of spelling, BBCB helped them to learn from their own mistakes. If appropriately used, this feature can play a significant role in enhancing the learners’ writing skills.

*[Positive emotion: Enjoyment] BBCB is really amazing. [Reason: Technological affordances] It can correct spelling when you write, and you can share videos, and ask questions directly to the teacher even after the lecture, there many other advantages that I like (Student C).*

More interestingly, one of the participants mentioned that using BBCB enhanced his motivation to study and engage in enhancing his writing skills:

*[Positive emotion: Attention] To be honest, I want to be recognized by the teacher [Reason: Assistance] so that he could help me and increase my marks in exam (Student C).*

When the same student was asked about the teacher’s interaction with students through BBCB, he said:

*[Positive emotion: Excitement] you feel really excited when the teacher sends you feedback in BBCB. [Reason: Motivation] Especially when he writes or says my name in front of everyone (Student C).*

Taken together, the findings discussed in this section suggest that using BBCB provide ample opportunities for learners to stay motivated and develop their writing skills. Writing in BBCB is a type of writing activity by itself. Activities such as writing questions, comments, feedback, and reading other learners’ comments, commenting on them, and reflecting on them help to improve writing skills as they do engage learners in the act of writing.

### Learners’ achievements

Although, the learners’ perceptions toward BB and BBCB were perceived to be positive, the implementation of BBCB did not affect the learners’ achievements. As shown in Table 7, results showed no significant differences between the learners’ achievements in the two semesters when either BBCB or FTF were implemented. In the FTF writing test the students achieved (M = 72.8889, Std = 13.09642), and in the BBCB, they scored (*M* = 67.9048, Std = 15.33918). However, the difference in the students’ achievement in the FTF test was not significant (Sig. = 0.287). In addition, contrary to the expected outcomes, the results showed that the learners who studied in FTF environment performed better than those who studied through BB. This finding can be attributed to the current lack of strategies related to the effective implementation of the BBCB platform into the EFL teaching process. Several students mentioned that, when using BB, they did not actually feel that they were in a real learning environment. For instance, one of the students said:

**TABLE 7:** Students’ achievements in Blackboard (BB) and face-to-face (FTF) classroom settings.

Variables	*N*	Mean	Std. deviation	*t*	Sig. (2-tailed)
F2F	58	72.8889	13.09642	1.081	0.287
BBCB	58	67.9048	15.33918

*[Negative emotion: Boring (lack of engagement)] Classes on blackboard do not make you feel that you are in a real learning environment, and there is no seriousness. [Reason: Lack of teacher’s control] Learners have freedom of doing other things at the same time, but it does not happen in face-to-face classes, where teacher can notice you and make you alert (Student D).*

Another explanation for the lack of a positive effect of using BBCB on the study participants’ achievements is that, for students, the sudden switch and unfamiliarity with BB technology in learning and teaching, could add to stress and anxiety and might have resulted in students’ lower academic performance. Yet, considering that the use of BB and BBCB was generally positively perceived by the participants, implementing appropriate teaching strategies using BBCB should be given a fair chance to enhance learners’ achievements.

## Discussion

Drawing on the sociocultural perceptive, this study investigated EFL learners’ perceptions of and attitudes to BB and the influence that learners’ emotion, elicited by online learning with BBCB, has on students’ collaborative writing and achievements. With regard to the first research question (“What are Saudi EFL tertiary learners’ perceptions on studying collaborative writing though Blackboard?”), the results revealed that, due to BBCB’s affordances that facilitate learners’ positive emotions and thus engage them in collaborative writing, the Saudi EFL students preferred to use BB to collaborate on various writing and communication tasks. Furthermore, the findings also showed that the learners very positively perceived BBCB as a platform for English learning. These findings are consistent with previous research on learners’ attitudes to and perceptions of BB use in the Saudi context (Almekhlafy, 2020; Elzainy et al., 2020; Almelhi, 2021). Due to the platform’s multimodal capabilities, and its features that are easy to use, and time-saving, BBCB created a perfect virtual zone where the students could feel comfortable while learning. This suggests that the more affordances a technology offers, the more it enhances the learners’ positive emotions toward it and raises the learners’ chances to engage in the learning process. Indeed, as demonstrated in several previous studies, technological affordances of online platforms that facilitate learning play a crucial role in engaging the students in various learning modalities, including collaborative writing (Li and Kim, 2016; Sobko et al., 2020).

As for the second research question (“To what extent can learners’ emotion influence collaborative writing through Blackboard and Chatbox?”), the results revealed that the students’ positive emotions toward BBCB fostered their frequent use of the platform to enhance their writing skills. In line with this finding, several previous studies on similar platforms, such as Wiki and other web-based platforms, found that affordances of learning platforms positively enhance collaborative learning (e.g., Onrubia and Engel, 2009; Kessler et al., 2012; Stoddart et al., 2013; Li and Zhu, 2017a,b). In the data, one of the interesting findings was that BBCB provided an opportunity for some students to show the teacher that they are good at writing, which allowed them to experience positive emotions that, in turn, increased their engagement in learning writing. As noted by one of the participants, the teacher’s attention would make the students feel “excited” and enhance their chances of getting better marks, which is an important motivation for students. Indeed, as shown in several previous studies, there is a clear link between students’ goal (better marks) and their level of engagement. For instance, Cho (2017) found a link between learners’ goals and their interaction patterns in a web-based collaborative writing. In addition, it was also found that emotion is linked to motivation and that several components of emotion such as comfort, liking, and affordances could play a significant role in learners’ engagement in an online environment. Therefore, based on the results, it can be concluded that BBCB could play an essential part in facilitating learners’ emotion if these components of emotion were taken care of by utilizing suitable teaching strategies to promote learners’ engagement and aiding them in achieving their goals.

Finally, as concerns to the third research question (“To what extent can Blackboard and Chatbox enhance learners’ achievements as compared to face-to-face?”), several contextual constraints of using CCBC negatively influenced the students’ achievements. The study reported that students in FTF scored higher than BBCB, however, the difference was not significant. Overall, the learners’ performance in BB settings was slightly lower than in FTF settings. Some students reported that online learning failed to arouse their interest in lectures, while others complained about the lack of control and motivation in using BBCB, as well as some technical issues, such as connectivity problems. In addition, the sudden shift from FTF to BB learning modalities made many students feel anxious and insecure about the change. Similar contextual constraints associated with the use of online learning platforms were also noted in other studies conducted in the Saudi context, showing that some Saudi students were dissatisfied with online learning due to technical issues and due to their negative feelings toward BB as an online learning option (Al-Ahdal and Alqasham, 2020; Al-Jarf, 2020; Al-Nofaie, 2020). For instance, Chen et al. (2013) found that, in online learning, the teacher’s failure to select appropriate writing topics negatively influenced students’ emotions. Similarly, learners’ negative emotion to the sudden shift from FTF to online learning was also reported by De Oliveira (2011) who argued that online learning should seek to establish robust social interaction between the students and the teacher, such as compliments and rapport, which can help to check anxiety. Therefore, in order to achieve sustainable implementation and enhanced results with BBCB, EFL instructors should be well prepared for the use of such educational technologies and be innovative in their use of this teaching methodology to cope with the change.

## Conclusion

Based on the results of the present study, the following conclusions can be formulated. First, it was found that the learners had predominantly positive perceptions of and attitudes toward BB and BBCB, and that the platform provided them with the affordances to overcome some contextual constraints. Specifically, the results revealed that the study participants’ use of the BBCB platform was associated with positive emotions toward this educational technology. Second, data analysis suggested the platform facilitated learners’ communication with the teacher, which increased their motivation to achieve their educational goals. However, the results showed that BBCB could not improve the students’ academic achievements as compared to the FTF learning modality. The factors that could have influenced this outcome may be related to the teacher’s poor preparation for the use of this online educational technology, which adversely affected some of the learners’ emotion associated with the use of BB. This sentiment is perhaps best captured in one of the interviewed students’ statements that “Blackboard is just the temporary platform to use and it is not forever; it has many options that access the work faster, but it cannot be used for teaching always” (Student D). Notwithstanding, due to its great technological features, Blackboard has an enormous potential in terms of improving English learning. In order to realize this potential, higher education institutions should provide necessary training for teachers that would allow language instructors to become more creative and more effectively use the functions of this educational technology.

### Recommendations

Based upon the findings, it is recommended that the BB system should be improved to address pending technological concerns, such as poor voice quality and slow internet connection. At the same time, institutions should be aware that learner performance is a dynamic process, and that a straightforward adoption of pedagogies, modes, or media cannot ensure the best learning outcomes. These require careful prior research and evaluation before being implemented in real educational settings. Another recommendation is that the BB learning system should be equipped with further functions beyond simple spelling check, such as checks for grammar and sentence structure.

These findings are of great significance to English language instructors and curriculum designers. Taking learners’ emotions into account when designing teaching materials or executing them in class would undoubtedly consolidate students’ motivation to learn in a friendly environment, and keep them involved in the learning process.

### Limitations and implications

The present study has several limitations. First, this study was conducted in a specific sociocultural context (Qassim region), which has its cultural and traditional peculiarities. Second, the sample was limited to male EFL learners from the Department of English Language and Translation, Methnab, Qassim University, Saudi Arabia. The third limitation of the present investigation is the suddenness with which the students and the teacher were moved from FTF learning to online learning through BB. At the time of this shift, most students and teachers did not possess the necessary expertise to use and operate the BBCB platform. These three limitations compromised the generalizability of the results.

Despite these limitations, the results provide important implications for improving collaborative writing in virtual classroom settings. Specifically, the results suggest that, along with focusing on students’ knowledge building in collaborative writing, teachers should also be more innovative in their teaching styles and consider their students’ emotional experiences, such as the changes in their enjoyment experience (Poehner and Swain, 2016; Dewaele et al., 2019). Given the dynamic nature of emotions, teachers should always strive to maintain their students’ enjoyment during the learning process. In addition to extensive use of technological features of the BB platform, teachers should seek for ways to enhance learners’ emotional involvement in collaborative writing. Among others, this could be achieved through offering students more interesting activities or topics and less structure in online educational settings.

## Data availability statement

The original contributions presented in this study are included in the article/Supplementary material, further inquiries can be directed to the corresponding author.

## Ethics statement

Ethical review and approval was not required for the study on human participants in accordance with the local legislation and institutional requirements. Written informed consent from the patients/participants or patients/participants legal guardian/next of kin was not required to participate in this study in accordance with the national legislation and the institutional requirements.

## Author contributions

FA: conceptualization, methodology, project administration, supervision, validation, writing – original draft preparation, investigation, supervision, and writing – review and editing.

## Footnotes

1 The first two digits (42) refer to the year, i.e., year 1442 in Islamic calendar, while the third digit 2 refers to the second semester (422).
