# Supplementary material for: Investigating English as a foreign language learners’ perceptions, emotions, and performance during online collaborative writing
Source: Front Psychol. 2022 Sep 14;13:954011. doi: 10.3389/fpsyg.2022.954011 (PMC9521550; doi:10.3389/fpsyg.2022.954011)
Supplement: Supplementary file 1 [file Data_Sheet_1.docx]

**Appendix A: Questionnaire**

| **No** | **Item** | | | | **Strongly Disagree** | | **Disagree** | | | **Neutral** | | | | **Agree** | | **Strongly Agree** | | **Mean** | | | **Std. deviation** |
| --- | --- | --- | --- | --- | --- | --- | --- | --- | --- | --- | --- | --- | --- | --- | --- | --- | --- | --- | --- | --- | --- |
| **General perception on blackboard Learning Management System**  **Positive Perception** | | | | | | | | | | | | | | | | | | | | | |
| **1** | I like to study on blackboard | | | | **3** | | **4** | | | **17** | | | | **10** | | **15** | | 3.61 | | | 0.73 |
|  |  |  |  |  | 6.12% | | 8.16% | | | 34.69% | | | | 20.41% | | 30.61% | |  |  |  |  |
| **2** | Blackboard helps me in many ways | | | | **1** | | **4** | | | **15** | | | | **13** | | **16** | | 3.73 | | | 1.18 |
|  |  |  |  |  | 2.04% | | 8.16% | | | 30.61% | | | | 26.53% | | 32.65% | |  |  |  |  |
| **3** | I feel comfortable while studying on blackboard | | | | **1** | | **4** | | | **7** | | | | **14** | | **23** | | 4.04 | | | 1.20 |
|  |  |  |  |  | 2.04% | | 8.16% | | | 14.29% | | | | 28.57% | | 46.94% | |  |  |  |  |
| **4** | Blackboard is like real classroom | | | | **14** | | **11** | | | **8** | | | | **12** | | **4** | | 2.61 | | | 1.35 |
|  |  |  |  |  | 28.57% | | 22.45% | | | 16.33% | | | | 24.49% | | 8.16% | |  |  |  |  |
| **5** | I enjoy learning through blackboard | | | | **4** | | **5** | | | **16** | | | | **14** | | **10** | | 3.40 | | | 1.20 |
|  |  |  |  |  | 8.16% | | 10.20% | | | 32.65% | | | | 28.57% | | 20.41% | |  |  |  |  |
| **6** | I get everything on blackboard | | | | **0** | | **12** | | | **12** | | | | **10** | | **15** | | 3.32 | | | 1.53 |
|  |  |  |  |  | 0.00% | | 24.49% | | | 24.49% | | | | 20.41% | | 30.61% | |  |  |  |  |
| **7** | Blackboard is easy to use | | | | **0** | | **2** | | | **1** | | | | **16** | | **30** | | 4.46 | | | 0.89 |
|  |  |  |  |  | 0.00% | | 4.08% | | | 2.04% | | | | 32.65% | | 61.22% | |  |  |  |  |
| **8** | It’s easy to upload homework or assignment on blackboard | | | | **0** | | **4** | | | **8** | | | | **9** | | **28** | | 4.16 | | | 1.21 |
|  |  |  |  |  | 0.00% | | 8.16% | | | 16.33% | | | | 18.37% | | 57.14% | |  |  |  |  |
| **9** | Blackboard saves time | | | | **1** | | **1** | | | **1** | | | | **12** | | **34** | | 4.57 | | | 0.81 |
|  |  |  |  |  | 2.04% | | 2.04% | | | 2.04% | | | | 24.49% | | 69.39% | |  |  |  |  |
| **10** | The blackboard learning tools are helpful to me | | | | **2** | | **3** | | | **9** | | | | **16** | | **19** | | 3.93 | | | 1.14 |
|  |  |  |  |  | 4.08% | | 6.12% | | | 18.37% | | | | 32.65% | | 38.78% | |  |  |  |  |
| **11** | I do not hesitate asking questions to the teacher on blackboard | | | | **1** | | **3** | | | **8** | | | | **10** | | **27** | | 4.16 | | | 1.16 |
|  |  |  |  |  | 2.04% | | 6.12% | | | 16.33% | | | | 20.41% | | 55.10% | |  |  |  |  |
| **12** | I can easily download the learning material shared by the teacher | | | | **1** | | **0** | | | **12** | | | | **17** | | **19** | | 4.10 | | | 0.84 |
|  |  |  |  |  | 2.04% | | 0.00% | | | 24.49% | | | | 34.69% | | 38.78% | |  |  |  |  |
| **13** | The learning material shared on blackboard is very helpful and clear to me | | | | **3** | | **3** | | | **7** | | | | **17** | | **19** | | 3.93 | | | 1.16 |
|  |  |  |  |  | 6.12% | | 6.12% | | | 14.29% | | | | 34.69% | | 38.78% | |  |  |  |  |
| **14** | I like the white screen shared by the teacher because it makes me feel like sitting in a real classroom | | | | **5** | | **5** | | | **12** | | | | **13** | | **14** | | 3.53 | | | 1.29 |
|  |  |  |  |  | 10.20% | | 10.20% | | | 24.49% | | | | 26.53% | | 28.57% | |  |  |  |  |
| **15** | I can access the lecture recordings at any time and get the benefit from them | | | | **0** | | **2** | | | **4** | | | | **10** | | **33** | | 4.46 | | | 0.95 |
|  |  |  |  |  | 0.00% | | 4.08% | | | 8.16% | | | | 20.41% | | 67.35% | |  |  |  |  |
| **General perception on blackboard Learning Management System**  **Negative perception** | | | | | | | | | | | | | | | | | | | | | |
| **16** | I face problems while logging in to the blackboard | **9** | | **12** | | | | **12** | | | **16** | | **0** | | 2.75 | | | | 1.33 | | |
|  |  | 18.37% | | 24.49% | | | | 24.49% | | | 32.65% | | 0.00% | |  |  |  |  |  |  |  |
| **17** | I find blackboard difficult to use | **22** | | **16** | | | | **7** | | | **3** | | **1** | | 2.00 | | | | 0.95 | | |
|  |  | 44.90% | | 32.65% | | | | 14.29% | | | 6.12% | | 2.04% | |  |  |  |  |  |  |  |
| **18** | I face internet and connectivity issues | **17** | | **6** | | | | **8** | | | **13** | | **5** | | 2.87 | | | | 1.23 | | |
|  |  | 34.69% | | 12.24% | | | | 16.33% | | | 26.53% | | 10.20% | |  |  |  |  |  |  |  |
| **19** | Blackboard doesn’t work if the internet is slow | **8** | | **5** | | | | **12** | | | **14** | | **10** | | 3.32 | | | | 1.26 | | |
|  |  | 16.33% | | 10.20% | | | | 24.49% | | | 28.57% | | 20.41% | |  |  |  |  |  |  |  |
| **20** | I face problems in downloading the course contents | **17** | | **14** | | | | **11** | | | **5** | | **2** | | 2.26 | | | | 1.11 | | |
|  |  | 34.69% | | 28.57% | | | | 22.45% | | | 10.20% | | 4.08% | |  |  |  |  |  |  |  |
| **21** | I face difficulty in accessing the lecture recording | **21** | | **18** | | | | **5** | | | **4** | | **1** | | 1.95 | | | | 0.99 | | |
|  |  | 42.86% | | 36.73% | | | | 10.20% | | | 8.16% | | 2.04% | |  |  |  |  |  |  |  |
| **22** | I like to study in a real classroom rather than on blackboard | **9** | | **5** | | | | **12** | | | **7** | | **16** | | 3.40 | | | | 1.38 | | |
|  |  | 18.37% | | 10.20% | | | | 24.49% | | | 14.29% | | 32.65% | |  |  |  |  |  |  |  |
| **23** | Blackboard is time-consuming | **22** | | **18** | | | | **5** | | | **3** | | **1** | | 1.91 | | | | 0.95 | | |
|  |  | 44.90% | | 36.73% | | | | 10.20% | | | 6.12% | | 2.04% | |  |  |  |  |  |  |  |
| **24** | My knowledge about blackboard is very limited | **18** | | **13** | | | | **8** | | | **8** | | **2** | | 2.34 | | | | 1.16 | | |
|  |  | 36.73% | | 26.53% | | | | 16.33% | | | 16.33% | | 4.08% | |  |  |  |  |  |  |  |
| **25** | Classes on blackboard are sometimes boring | **8** | | **9** | | | | **10** | | | **13** | | **9** | | 3.10 | | | | 1.38 | | |
|  |  | 16.33% | | 18.37% | | | | 20.41% | | | 26.53% | | 18.37% | |  |  |  |  |  |  |  |
| **Perception on blackboard’s chat box in enhancing writing skill** | | | | | | | | | | | | | | | | | | | | | |
| **26** | I like writing in blackboard’s chat box | **3** | **6** | | | **14** | | | **13** | | | **13** | | | | | 3.48 | | | 1.29 | |
|  |  | 6.12% | 12.24% | | | 28.57% | | | 26.53% | | | 26.53% | | | | |  |  |  |  |  |
| **27** | Blackboard’s chat box corrects my spelling mistakes | **4** | **13** | | | **19** | | | **3** | | | **10** | | | | | 2.85 | | | 1.42 | |
|  |  | 8.16% | 26.53% | | | 38.78% | | | 6.12% | | | 20.41% | | | | |  |  |  |  |  |
| **28** | I can freely write in the blackboard’s chat box | **1** | **5** | | | **8** | | | **14** | | | **21** | | | | | 3.91 | | | 1.27 | |
|  |  | 2.04% | 10.20% | | | 16.33% | | | 28.57% | | | 42.86% | | | | |  |  |  |  |  |
| **29** | I prefer writing in the chat box than speaking in the microphone | **6** | **15** | | | **14** | | | **6** | | | **8** | | | | | 2.71 | | | 1.44 | |
|  |  | 12.24% | 30.61% | | | 28.57% | | | 12.24% | | | 16.33% | | | | |  |  |  |  |  |
| **30** | I have no fear of mistakes and errors while writing in the chat box | **1** | **5** | | | **14** | | | **14** | | | **15** | | | | | 3.67 | | | 1.23 | |
|  |  | 2.04% | 10.20% | | | 28.57% | | | 28.57% | | | 30.61% | | | | |  |  |  |  |  |
| **31** | I write longer sentences in the chat box | **0** | **12** | | | **14** | | | **7** | | | **16** | | | | | 3.30 | | | 1.54 | |
|  |  | 0.00% | 24.49% | | | 28.57% | | | 14.29% | | | 32.65% | | | | |  |  |  |  |  |
| **32** | I write questions in the chat box when I face problems | **1** | **2** | | | **9** | | | **17** | | | **20** | | | | | 4.06 | | | 1.02 | |
|  |  | 2.04% | 4.08% | | | 18.37% | | | 34.69% | | | 40.82% | | | | |  |  |  |  |  |
| **33** | I write comments in the chat box | **1** | **3** | | | **9** | | | **16** | | | **20** | | | | | 4.00 | | | 1.11 | |
|  |  | 2.04% | 6.12% | | | 18.37% | | | 32.65% | | | 40.82% | | | | |  |  |  |  |  |
| **34** | I write small paragraphs in the chat box on a topic given by the teacher | **1** | **7** | | | **16** | | | **14** | | | **11** | | | | | 3.42 | | | 1.27 | |
|  |  | 2.04% | 14.29% | | | 32.65% | | | 28.57% | | | 22.45% | | | | |  |  |  |  |  |
| **35** | I write paragraphs and essays and submit as homework in the chat box | **4** | **12** | | | **15** | | | **6** | | | **12** | | | | | 3.04 | | | 1.48 | |
|  |  | 8.16% | 24.49% | | | 30.61% | | | 12.24% | | | 24.49% | | | | |  |  |  |  |  |
| **36** | I watch other students’ comments and questions in the chat box and learn from them | **0** | **0** | | | **5** | | | **20** | | | **24** | | | | | 4.38 | | | 0.67 | |
|  |  | 0.00% | 0.00% | | | 10.20% | | | 40.82% | | | 48.98% | | | | |  |  |  |  |  |
| **37** | Teacher corrects my sentences in the chat box | **2** | **4** | | | **9** | | | **15** | | | **19** | | | | | 3.87 | | | 1.21 | |
|  |  | 4.08% | 8.16% | | | 18.37% | | | 30.61% | | | 38.78% | | | | |  |  |  |  |  |
| **38** | Teacher gives me feedback and I correct my mistakes in the chat box | **1** | **2** | | | **14** | | | **12** | | | **20** | | | | | 3.95 | | | 1.07 | |
|  |  | 2.04% | 4.08% | | | 28.57% | | | 24.49% | | | 40.82% | | | | |  |  |  |  |  |
| **39** | I find writing in the chat box is easier than writing on a paper | **4** | **4** | | | **10** | | | **15** | | | **16** | | | | | 3.71 | | | 1.24 | |
|  |  | 8.16% | 8.16% | | | 20.41% | | | 30.61% | | | 32.65% | | | | |  |  |  |  |  |
| **40** | I can watch recording at any time and correct my mistakes in paragraphs or essays | **1** | **4** | | | **9** | | | **9** | | | **26** | | | | | 4.06 | | | 1.24 | |
|  |  | 2.04% | 8.16% | | | 18.37% | | | 18.37% | | | 53.06% | | | | |  |  |  |  |  |
| **41** | Blackboard’s chat box helps me a lot in improving my writing skill | **2** | **5** | | | **11** | | | **13** | | | **18** | | | | | 3.75 | | | 1.28 | |
|  |  | 4.08% | 10.20% | | | 22.45% | | | 26.53% | | | 36.73% | | | | |  |  |  |  |  |
| **42** | I feel proud when I see my teachers’ positive comments and feedback on my writing in the chat box | **0** | **0** | | | **11** | | | **15** | | | **23** | | | | | 4.24 | | | 0.80 | |
|  |  | 0.00% | 0.00% | | | 22.45% | | | 30.61% | | | 46.94% | | | | |  |  |  |  |  |
| **43** | My writing skill is improved because of the use of blackboard’s chat box | **8** | **3** | | | **14** | | | **8** | | | **16** | | | | | 3.53 | | | 1.27 | |
|  |  | 16.33% | 6.12% | | | 28.57% | | | 16.33% | | | 32.65% | | | | |  |  |  |  |  |
| **44** | I find myself as a better writer than I was before | **4** | **5** | | | **11** | | | **14** | | | **15** | | | | | 3.61 | | | 1.28 | |
|  |  | 8.16% | 10.20% | | | 22.45% | | | 28.57% | | | 30.61% | | | | |  |  |  |  |  |
| **45** | Blackboard’s chat box is a good tool to improve writing skill in the virtual classrooms. | **3** | **5** | | | **15** | | | **10** | | | **16** | | | | | 3.59 | | | 1.28 | |
|  |  | 6.12% | 10.20% | | | 30.61% | | | 20.41% | | | 32.65% | | | | |  |  |  |  |  |
| N=49 | | | | | | | | | | | | | | | | | | | | | |

**Appendix B:** Learners’ achievements in BB compared to face-to-face classroom settings

| **No** | **Standard Score/Grade** | | **Learners’ achievements** | |
| --- | --- | --- | --- | --- |
|  | **Score** | **Grade** | **In BB setting** | **In face-to-face setting** |
| 1 | 95-100 | A+ | 00.00% | 00.00% |
| 2 | 90-94 | A | 09.52% | 11.11% |
| 3 | 85-89 | B+ | 04.76% | 05.55% |
| 4 | 80-84 | B | 14.28% | 27.77% |
| 5 | 75-79 | C+ | 09.52% | 16.66% |
| 6 | 70-74 | C | 09.52% | 00.00% |
| 7 | 65-69 | D+ | 14.28% | 22.22% |
| 8 | 60-64 | D | 19.04% | 05.55% |
| 9 | 0-59 | F | 19.04% | 11.11% |
